# Neurophysiological Correlates of Expert Knowledge: An Event-Related Potential (ERP) Study about Law-Relevant Versus Law-Irrelevant Terms

**DOI:** 10.3390/brainsci14101029

**Published:** 2024-10-17

**Authors:** Peter Walla, Stefan Kalt, Konrad Lachmayer

**Affiliations:** 1 Freud CanBeLab, Faculty of Psychology, Sigmund Freud Private University, Freudplatz 1, 1020 Vienna, Austria; stefan.kalt@sfu.ac.at; 2 Faculty of Medicine, Sigmund Freud Private University, Freudplatz 3, 1020 Vienna, Austria; 3 School of Psychology, Newcastle University, University Drive, Newcastle, NSW 2308, Australia; 4 Faculty of Law, Sigmund Freud Private University, Lassallestrasse 3, 1020 Vienna, Austria; konrad.lachmayer@jus.sfu.ac.at

**Keywords:** electroencephalography, event-related potential (ERP), expert knowledge, non-conscious processing

## Abstract

Background: The evaluation of evidence, which frequently takes the form of scientific evidence, necessitates the input of experts in relevant fields. The results are presented as expert opinions or expert evaluations, which are generally accepted as a reliable representation of the facts. A further issue that remains unresolved though is the process of evaluating the expertise and knowledge of an expert in the first instance. In general, earned certificates, grades and other objective criteria are typically regarded as representative documentation to substantiate an expert status. However, there is a possibility that these may not always be sufficiently representative. Objectives: The goal of the present study was to provide evidence that the neural processing of law-relevant and law-irrelevant terms varies significantly between participants who have received training in the field of law (experts) and those who have not (novices). Methods: To this end, changes in brain activity were recorded via electroencephalography (EEG) during visual presentations of terms belonging to five different categories (fake right, democracy, filler word, basic right and rule of law). Event-related potentials (ERPs) were subsequently averaged for each category and subjected to statistical analysis. Results: The results clearly demonstrate that participants trained in law processed fake rights and filler words in a similar manner. Furthermore, both of these conditions elicited different levels of brain activity compared to all law-relevant terms. This was not the case in participants who had not received legal training. The brains of untrained participants processed all five term categories in a strikingly similar manner. In light of prior knowledge regarding language processing, the primary focus was on two distinct electrode locations: one in the left posterior region, and the other in the left frontal region. In both locations, the most prominent differences in brain activity elicited by the aforementioned term categories in law-trained participants occurred approximately 450 milliseconds after stimulus onset. The results were further corroborated by a repeated-measures ANOVA and subsequent *t*-tests, which also demonstrated the absence of this effect in law-untrained participants. Conclusions: The findings of this study provide empirical evidence that brain activity measurements, in particular ERPs, can be used to distinguish between experts trained in a specific field of expertise and novices in that field. Such findings have the potential to facilitate objective assessments of expertise, enabling comparisons between experts and novices that extend beyond traditional criteria such as qualifications and experience. Instead, individuals can be evaluated based on their cognitive processes, as observed through brain activity.

## 1. Introduction

The question of whether French or American wines are superior is one that is best possibly answered by experts in the field. Indeed, in 1976, a blind wine tasting competition was held in Paris, named in reference to the Greek myth known as the “Judgement of Paris” [1]. In the 1970s, French wines were considered the best in the world, but even though among the eleven expert judges nine were from France, American wines won the race. Thirty years later, in 2006, a blind re-tasting took place with several French tasters, who participated in the original competition. American wines won again, with a Californian Cabernet in first place. While the specific result of this competition is not a primary focus of this study, the role of experts and their difference from novices in general provides a foundation for our investigation. In 1955, Gibson & Gibson [2] were already interested in differences between trained wine experts and untrained wine tasters. Later, psychological investigations tried to find connections between sensory and cognitive processes related to wine tasting [3,4,5]. While sensory processes simply feed the brain in a bottom-up manner with environmental information, it is following cognitive processing that forms the basis for decisions [6]. With respect to wine, it is the combination of sensory bottom-up and knowledge-based, cognitive top-down processes that reflects the respective expertise [7,8], but what does a comparison between experts and novices look like in a purely knowledge-based field such as a theoretical tertiary education discipline?

To reach expert-level knowledge in a distinct discipline, one obviously needs a respective education that includes dedicated efforts of consistent learning, practice and relevant experience. Experts deliver critical decisions in many areas and teach their knowledge and expertise to the next generations. They are essential for evaluations and decision-making in research and development, diagnosis and treatment in the healthcare sector, risk management, product design and marketing, to name just a few. Crucially, experts are not born, they are made [9,10]. This conclusion is based on rigorous empirical research that looked at exceptional performance within various disciplines [11,12]. Comparing experts and non-experts is a growing field of research [13], and expert knowledge is indeed a result of intense efforts put into consistent learning, practice and respective exposure to gain experience. The key characteristic that sets experts apart from novices is an in-depth understanding in their field beyond superficial knowledge. Besides additional real-life application experience, experts are able to develop intuition-based decision-making allowing them to provide accurate judgements, often without being able to explicitly explain on what basis they actually came to their conclusion. From this notion, the question arises of how to measure such expertise without simply checking the respective certificates, grades and professional, documented experience.

It is this question that forms the basis for the current study, which aims at providing neurophysiological evidence for expert knowledge on the basis of language processing. This research goal is grounded in the idea that only objective neurophysiology is able to get access to implicit information processing [14] that can be understood as a prerequisite for intuition-based expert-level decision-making. Before getting into any details, it is important to look at existing neuroscience literature regarding the topic of expert knowledge. In neuroscience, expert recognition of objects as visual inputs has long been a focus of interest [15]. Thereby, the far most researched neurophysiological correlate is the so-called N170 event-related potential (ERP) component [16]. This very solid negative ERP component at around 170 ms after face presentation onset is very robustly found at right occipito-temporal electrode locations [17]. The respective cortical region is referred to as the fusiform face area (FFA), named after the *Fusiform gyrus*, which has been found as the neural generator for this component [18]. However, Harel and colleagues [15] started to question such a face-centric view and rather believe expert-related activity to be found throughout the visual cortex (not just at the FFA), with behavioural expertise strongly correlating with neural responses even in parietal and prefrontal cortical regions. Anyway, even though the human brain has been shown to be an expert in face processing, it remains unclear, to the best of our knowledge, if it is possible to find neurophysiological correlates that allow for a distinction between experts and non-experts (novices) regarding discipline-specific language processing (terminology). In other words, the current study aimed to focus on expert knowledge as in theoretical knowledge-based expertise in a distinct high-level tertiary education subject in combination with a high temporal resolution brain imaging tool that is able to capture even short-lasting brain activity differences. On the basis of the above-mentioned face-specific ERP component, it was decided to use electroencephalography (EEG) as the neurophysiological method of investigation. EEG and particularly generated ERPs represent an excellent tool to measure brain activity changes in the millisecond range [19,20]. While the spatial resolution of EEG is not as good as that of other brain imaging methods, it is the very high temporal resolution that allows for describing the smallest functional processing differences. With respect to the expert terminology to be investigated, we chose to focus on law-related expertise. This was largely based on recent literature that highlights the incomprehensible nature of the law language in general and of legal documents in particular [21]. The neurophysiological investigation of language processing in general has a long history. Due to the high temporal resolution of EEG, various ERP components have been described, each of which are related to distinct language aspects such as lexical, semantic, contextual and even prosodic aspects. Most importantly, left temporo-parietal and left frontal brain regions were found to be involved in language processing. In line with this existing knowledge, we formed the hypothesis that the processing of law-relevant versus law-irrelevant terms varies as a function of received law training versus no such training in left hemispheric temporo-parietal and/or frontal brain regions, as measured with EEG.

## 2. Materials and Methods

### 2.1. Participants

In total, 31 participants were invited to the Freud CanBeLab (Freud Cognitive and Affective Neuroscience and Behavior Lab) at Sigmund Freud Private University in Vienna (Austria). Their mean age was 31.06 (SD = 2.51) years. They were all right-handed and had no neuropathological history. Importantly, 17 participants were well trained in tertiary legal education (9 males), whereas 14 participants did not receive any legal or law training (8 males). The mean age in the law-trained group was 31.06 years (SD = 2.43), and the mean age in the law-untrained group was 31.07 years (SD = 2.60). All participants gave their informed consent and were told that they could withdraw from the study at any time without any negative consequences. The study design including all ethics-relevant details was submitted to the ethics committee of Sigmund Freud Private University, whose members gave their approval (approval code: UD39NGJTC28MFI90754).

### 2.2. Stimuli

Five term categories were selected, and respective term lists were created (25 terms per category) as stimuli to be visually presented on a computer monitor. The categories were “democracy” (e.g., referendum, Federal Council), “basic rights” (e.g., fair trial, freedom of expression), “rule of law” (e.g., legal protection, Constitutional Court), “fake rights” (e.g., card freedom, mixing right) and “filler words” (passport number, letterbox). Stimulus presentation was administered and controlled by the free open source software PsychoPy2 for Windows11 (Version: 2021.2.3) [22]. The same software was also used to send triggers to the EEG system in order to provide condition coding for later EEG and ERP data analysis.

### 2.3. Procedure

After arrival at the lab, the participants were introduced to the purpose of the study. They were given the informed consent form to sign if they agreed to participate. The actiCAP with 64 electrodes embedded (from Brain Products; Gilching, Germany) was applied and connected to an amplifier (see further details below). Before the recordings started, the participants were instructed to sit still and blink with their eyes only when they saw a fixation cross, but to avoid blinking during verbal presentations. Each term was presented twice (in random order; 25 presentations per category) for 300 ms in white letters on a black background on a computer monitor placed on a table in front of the participants, who sat on a comfortable chair. This was followed by a black screen for 1 s and a white fixation cross on a black background for 1 s with a final black screen for, again, 1 s. The eye-to-screen distance was about 0.7 m. All visual stimuli were presented so as to stimulate the foveal receptors only (no peripheral field stimulation). The participants were instructed to indicate via a button press whether the term they saw was a true legal term or not.

On average, law-untrained participants responded 83.6% correctly, and law-trained participants responded 93.3% correctly.

### 2.4. Electroencephalography (EEG)

For recording brain potential changes, a 64-channel actiCHamp Plus System from Brain Products (Gilching, Germany) with active electrodes embedded in an actiCAP connected to an amplifier was used. The amplifier was operated by a powerful lithium-ion battery pack. The brain potentials were sampled at a rate of 1 kHz (filtered: DC to 100 Hz). Impedance was kept equal to or below 10 kΩ. Cz was used as reference, and a mid-frontal position on the forehead was used as the ground electrode. Offline, all EEG data were down-sampled to 250 Hz, and a bandpass filter from 0.1 to 30 Hz was applied in preparation for following EEG data processing. Those data were then used to generate ERPs. For this purpose, 1.1 s long time windows (epochs) were cut out of the ongoing EEG recordings starting 100 ms before each trigger until 1 s after the trigger. Baseline correction was performed by using the 100 ms time period before each trigger. Finally, all epochs within each of the 5 term categories were averaged to generate ERPs. Those were then used to display term category-specific neurophysiological activity changes over time. All EEG data processing was conducted with the EEGDisplay (Version 6.4.9) software (by Ross Fulham).

### 2.5. Analyses

After generating ERPs for all five term categories for each participant, the software EEGDisplay was further used to export neurophysiological data for statistical analyses. For this purpose, all ERP data were again down-sampled to reduce the data volume. The result was 16 ms long time windows (averages across four 4 ms potential values). Those time windows were then subject to statistical analysis. At this point, it is important to mention that 2 specific electrode locations were selected for further analyses. Those were the left frontal electrode position FC5 and the left parietal electrode position P7. This selection was based on prior literature on relevant language processing [23].

First, repeated-measures Analysis of Variance (ANOVA) was applied for all time windows between 300 ms and 600 ms after stimulus onset. The following within-subject factors were defined: term (5 levels: “fake rights”, “democracy”, filler words”, “basic rights”, “rule of law”); and electrode (2 levels: FC5, P7). The only between-subject factor was group (2 levels: law-trained participants, law-untrained participants). Afterwards, for each time window showing a significant interaction of all three factors, *t*-tests were calculated to compare each possible pair of term condition separately for each group and for each electrode position. This was performed to provide detailed statistical results to be used for comparisons between single ERPs for both groups and all term conditions.

## 3. Results

### 3.1. Event-Related Potentials (ERPs) and Topographical Maps

**Figure 1:**
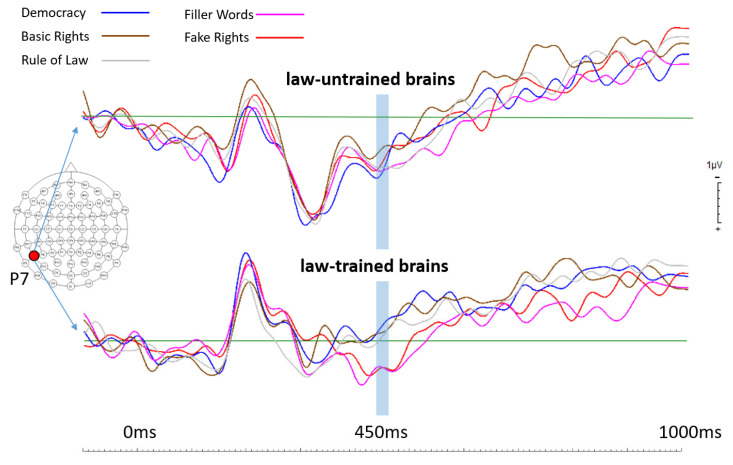
Event-related potentials (ERPs) calculated for all 5 term categories for the left parietal electrode position P7 in both groups (law-trained and law-untrained participants). Statistical analysis revealed that the time window around 450 ms post stimulus (from 443 ms to 459 ms after stimulus onset) showed the most dominant effects in law-trained participants. This time window is marked with a light blue bar across all ERPs. Note that in law-trained participants, both fake rights and filler words elicited similar brain activity levels (potentials) that differ in very similar ways from those of all 3 law-relevant terms that elicited very similar activities. This pattern of brain activities is totally absent in law-untrained participants.

**Figure 2:**
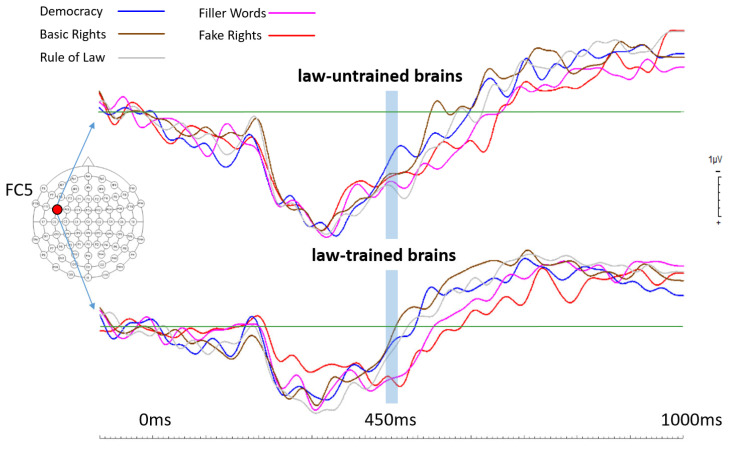
Event-related potentials (ERPs) calculated for all 5 term categories for the left frontal electrode position FC5 in both groups (law-trained and law-untrained participants). Statistical analysis revealed that the time window around 450 ms post stimulus (from 443 ms to 459 ms after stimulus onset) showed the most dominant effects in law-trained participants. This time window is marked with a light blue bar across all ERPs. Note that in law-trained participants both fake rights and filler words elicited similar brain activity levels (potentials) that differ in very similar ways from those of all 3 law-relevant terms that elicited very similar activities. This pattern of brain activities is totally absent in law-untrained participants. This finding resembles the one described for the left parietal electrode position P7, with just smaller activity differences (that also started a bit later) between fake rights and filler words and the rest of the term conditions.

**Figure 3:**
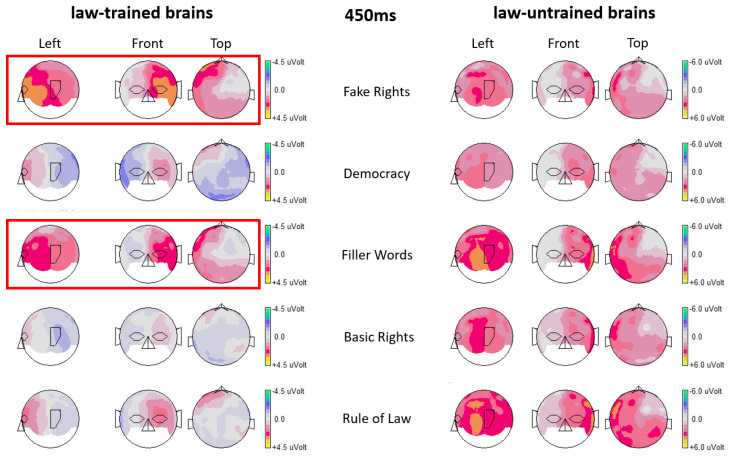
Topographical maps including data from all electrode locations created for all 5 term conditions for the time point 450 ms post stimulus and for both groups. Red boxes mark the topographies for the two critical term categories that elicited similar brain activity levels in law-trained brains (fake rights and filler words).

Overlaid ERPs from both participant groups generated for electrode position P7 showed a clear pattern of brain activity differences. In law-trained participants, starting at around 400 ms after stimulus onset, brain activities elicited by fake rights and filler words (both categories are not law-relevant) deviated from those elicited by all other conditions, which are all law-relevant categories. This grouping of brain activity levels in law-trained participants set apart language processing of law-relevant versus that of law-irrelevant terms. This brain activity pattern was totally absent in law-untrained participants (see Figure 1 for the respective ERPs generated for electrode position P7). The overlaid ERPs from both participants groups generated for electrode position FC5 showed a very similar brain activity pattern across all five term conditions when compared to the findings from electrode position P7 (see Figure 2). The only difference seemed to be somewhat smaller differences between brain activities elicited by law-relevant terms and those elicited by law-irrelevant terms in law-trained participants (and the differences also seemed to begin slightly later at FC5). Again, in law-untrained participants, no such effects were evident. Figure 3 shows topographical maps including data from all electrode locations.

In summary, the brains of law-trained participants seemed to clearly distinguish between law-relevant and law-irrelevant terms, whereas the brains of law-untrained participants did not.

### 3.2. Analytical Statistics

The analysis of variance (repeated-measures ANOVA) including the within-subject factors *term* (5 levels: fake rights, democracy, filler words, basic rights, rule of law) and *electrode* (2 levels: FC5 and P7) and the between-subject factor *group* (2 levels: law-trained and law-untrained subjects) resulted in a significant term*electrode*group interaction for the time interval from 443 ms to 459 ms (*p* = 0.047 (Greenhouse–Geisser-corrected), F = 2.795; partial eta-square η^2^ = 0.088). This medium-size significant ANOVA effect allowed for further and more detailed comparisons in the frame of paired-sample *t*-tests. Those were carried out for all possible pairwise comparisons of brain activity levels elicited by all term conditions in both groups for both selected electrode conditions and the time frame that showed the above-mentioned significant interaction of all factors (see Table 1 and Table 2). Those *t*-tests perfectly confirmed that the above-described differences only occurred in law-trained brains (Table 1), with only a few differences of other kinds in law-untrained brains (Table 2).

## 4. Discussion

The present study was meant to provide empirical, neurophysiological evidence that expert knowledge [24] on the basis of language processing [25] can be shown objectively by using ERPs. As mentioned in the introduction, ERPs are an excellent means of describing even the shortest functional differences in the human brain [26,27,28]. The obtained results now demonstrate very clearly that ERPs reflecting language processing of law-relevant versus law-irrelevant terms presented to law-trained and law-untrained participants allow for distinguishing between these two groups. In other words, ERPs enable a clear objective distinction between experts and novices.

As can be seen in Figure 1 and Figure 2, the main differences occurred in a time window roughly spanning from 400 ms to 600 ms after stimulus onset. The selected electrode locations represent crucial brain regions for language processing [23]. The middle temporal gyrus was found as the most likely candidate for storing lexical representations [23], and the effects that were found in the current study at electrode position P7 are thus best interpreted as lexical effects. Electrode position P7 also represents a brain region widely known as the Wernicke area [29]. This area is generally understood as the comprehension centre involved in comprehending written and spoken language. At this point, it is not clear whether the effects found in the current study at electrode position P7 are about differences in lexical and/or semantic language processing [30,31], but as pointed out by Davis and Johnsrude [32] and by Zekveld and colleagues [33], effects in that region are probably driven by top-down processing. This would make perfect sense, given that the brains of law-trained participants may use their law-related education to process law-relevant terms differently compared to the brains of law-untrained participants.

At first glance, the results of the study do not appear to be surprising at all. Lawyers are experts in legal terminology [34]; so, their brains will respond to legal terms differently from those of non-lawyers. They are also able to distinguish legal terms from fake terms, because they are trained to do so. In contrast, the brains of non-lawyers cannot distinguish legal terms from general language terms and cannot filter out fake terms. In this respect, the study confirmed existing general assumptions. Nevertheless, this result is already an important step towards a cognitive understanding of law as an expert language. It demonstrates expertise on a new and different level compared to traditional approaches, which analysed these differences in particular from a sociological or epistemological perspective [35,36].

It is of particular interest that the chosen terminology comes from constitutional law, whose terminology is more likely to be found in general public debates. While most of the terms chosen are not part of the common language (e.g., principle of legality), the sample also included terms such as Member of Parliament or Constitutional Court. The results show a clear difference between legally trained and non-legally trained brains with regard to constitutional terminology, including rights. No specific knowledge of rights was identified among non-legally trained people. This is confirmed by a sociological study on the Austrian population’s knowledge of their rights, which is significantly low [37].

In contrast, the category ‘democracy’ has gained a certain significance in the group of lay people, the reason for which does not seem to be clear. This could relate to a better general education about democracy and the possibility that lay people could be better educated regarding basic constitutional or legal concepts. This could create interesting perspectives in the field of popular knowledge about law [38].

The distinction of fake rights by law-trained rather than law-untrained participants illustrates that linguistic inventions are no different from ordinary language, which was not clear before the study. It is therefore noteworthy that not only is it evidently not legal terminology (for law-trained participants), but also it is part of ordinary language processing.

## 5. Future Research

As this study is a fundamental starting point for further research, many research questions and further fields of research can be identified. This research dealt with both law-trained and law-untrained people. As far as law-trained people are concerned, this refers to legal education [39]. While the study focused on individuals who not only were trained in law but also had been practicing law for a number of years, it would be interesting to see if the same effects can be observed with students who have just graduated from law school or who are still studying. Does it make a difference whether they are excellent, good, average or poor students? Since we are talking about the beginning of a legal career, it might also be worth comparing these results with those from lawyers at the end of their professional careers.

If one chooses a perspective relating to law-untrained people, the population’s knowledge of the law, in particular of their rights, proves to be an important topic. However, people’s knowledge of their rights differs considerably in various countries [40]. While studies in this context are based on sociological research [36], it would be possible through neuroscience to find out whether the results obtained in this study can be replicated in other countries and how they differ. In this respect, a new form of cognitive science study can complement existing studies in the social sciences. The understanding of the general population’s knowledge of the law can be deepened in this respect. When selecting the legal terminology, the focus was placed on constitutional law. It would be important to extend the selected terminology to other areas of law.

From the perspective of comparative law, many new questions arise. The first concerns the replication of the study in other countries. The study presented here focused on Austria and thus on the German language as it is represented in the Austrian legal system. This does not mean that the results would necessarily be the same in another German-speaking country such as Germany or Switzerland (the legal terminology would have to be adapted, as different countries have different legal terminologies). It is also very important to go beyond the German language, as the compound terms of the German language are very particular [41]. Comparative studies can also look at the role of popular knowledge on specific topics and the role of legal terminology in a particular legal system in general. Another possibility is to focus on the comparative scholars themselves and examine the extent to which they are able to acquire legal knowledge in another country [35].

With regard to the fundamental impact of the use of Artificial Intelligence (AI) in legal science and practice [42,43], neuroscientific studies may be relevant on the one hand for understanding legal thinking with regard to the training of AI systems. On the other hand, it could be crucial to see how AI changes people’s legal thinking, e.g., if expertise is lost as a result of AI being used by humans on an ongoing basis.

On a general level, expert language used in law can be compared to expert language in other fields such as medicine, engineering or philosophy. Does the concept of expert language work in the same way in all fields or is it different? The acquisition and representation of expert language in different subject areas could lead to a more general understanding of expert language and expertise. In addition, the methods of cognitive neuroscience could provide further insights into expert language and knowledge in general.

## 6. Limitations

First of all, we want to mention that the samples in our study were rather small, which is a common concern in studies like this. However, by applying standard statistical analysis methods significant effects were found, and given the small sample sizes, this means that the found effects are actually quite robust. Furthermore, the drawn conclusion that the current results are seen as neurophysiological correlates of expert knowledge might be far-fetched and should thus be treated with a certain amount of caution.

## 7. Conclusions

The study showed that law-trained people as experts process legal terminology different compared to law-untrained individuals. This first brain-based evidence confirmed what was already known about legal thinking. Furthermore, the applied neuroscientific method can of course only provide a small insight into the complex processes of legal thinking, which go far beyond legal terminology and its effects on brain potentials. Nevertheless, it is a starting point for reconsidering our understanding of legal thinking [44]. As the different research perspectives show, this research could initiate various forms of studies that will gain more practical relevance in terms of legal education, popular knowledge of law, comparative law and the role of AI systems in the field of law.

## Figures and Tables

**Table 1:** *t*-Test results for both selected electrodes and all possible comparisons for the law-trained group. Comparisons were carried out for the time frame from 443 to 459 ms after stimulus onset, because for this time frame, a repeated-measures ANOVA revealed a significant interaction between the factors *condition*, *electrode* and *group* (law-trained versus untrained subjects). The pattern of *t*-test results underlines the finding that in law-trained subjects, the two conditions “fake rights” and “filler words” elicited comparable brain activities, which both differed from those elicited by the rest of the conditions that among themselves elicited similar brain activity levels (significant *p*-values are marked in red). Note that this was true for both selected electrode locations.

Law-Trained Brains	*t*-Test Pairs	T	df	*p*-Value
Electrode FC5 at 443 to 459 ms	Fake right/Democracy	1.777	17	0.047
	Fake right/Basic right	4.181	17	≤0.001
	Fake right/Rule of law	1.802	17	0.045
	Fake right/Filler word	−0.032	17	0.488
	Filler word/Democracy	−2.138	17	0.024
	Filler word/Basic right	3.799	17	≤0.001
	Filler word/Rule of law	1.929	17	0.035
	Democracy/Basic right	0.716	17	0.242
	Democracy/Rule of law	−0.317	17	0.378
	Basic right/Rule of law	−1.376	17	0.095
Electrode P7 at 443 to 459 ms	Fake right/Democracy	3.294	17	0.002
	Fake right/Basic right	3.608	17	0.001
	Fake right/Rule of law	2.240	17	0.019
	Fake right/Filler word	−0.430	17	0.336
	Filler word/Democracy	−3.664	17	≤0.001
	Filler word/Basic right	4.675	17	≤0.001
	Filler word/Rule of law	4.369	17	≤0.001
	Democracy/Basic right	−0.686	17	0.251
	Democracy/Rule of law	−0.601	17	0.278
	Basic right/Rule of law	−0.154	17	0.440

**Table 2:** *t*-Test results for both selected electrodes and all possible comparisons for the law-untrained group. Comparisons were carried out for the time frame from 443 to 459 ms after stimulus onset, because for this time frame, a repeated-measures ANOVA revealed a significant interaction between the factors *condition*, *electrode* and *group* (law-trained versus untrained subjects). The pattern of *t*-test results underlines the finding that in law-untrained subjects, almost no differences in brain activity levels were found. Only a few differences occurred, but the clear pattern of the results obtained for the law-trained group was completely absent (significant *p*-values are marked in red). Note that this was true for both selected electrode locations.

Law-Untrained Brains	*t*-Test Pairs	T	df	*p*-Value
Electrode FC5 at 443 to 459 ms	Fake right/Democracy	1.625	12	0.065
	Fake right/Basic right	1.012	12	0.166
	Fake right/Rule of law	0.177	12	0.431
	Fake right/Filler word	−0.406	12	0.346
	Filler word/Democracy	−3.872	12	0.001
	Filler word/Basic right	0.848	12	0.206
	Filler word/Rule of law	0.594	12	0.282
	Democracy/Basic right	−0.739	12	0.237
	Democracy/Rule of law	−1.971	12	0.036
	Basic right/Rule of law	−0.375	12	0.357
Electrode P7 at 443 to 459 ms	Fake right/Democracy	−0.410	12	0.345
	Fake right/Basic right	1.403	12	0.093
	Fake right/Rule of law	−0.277	12	0.393
	Fake right/Filler word	−1.087	12	0.149
	Filler word/Democracy	−0.694	12	0.149
	Filler word/Basic right	1.850	12	0.045
	Filler word/Rule of law	0.568	12	0.290
	Democracy/Basic right	1.336	12	0.103
	Democracy/Rule of law	−0.068	12	0.473
	Basic right/Rule of law	−0.921	12	0.188

## Data Availability

The data presented in this study are available on request from the corresponding author.
